# ECCO_2_R, a french national survey

**DOI:** 10.1186/2197-425X-3-S1-A679

**Published:** 2015-10-01

**Authors:** B Deniau, JD Ricard, J Messika, D Dreyfuss, S Gaudry

**Affiliations:** Hopital Louis Mourier, Assistance Publique Hopitaux de Paris, Intensive Care Unit, Colombes, France

## Introduction

ECCO_2_R (extracorporeal dioxide carbon removal) is an extracorporeal decarboxylation technology described in 1978^1^. Physiological studies showed that 50% of produced CO2 were eliminated^2^. Potential indications are: ultra-protective mechanical ventilation (MV) for acute respiratory distress syndrome (ARDS)^3^ and hypercapnic patients at risk of failure of noninvasive ventilation (NIV)^4^. Because of the lack of scientific evidence, ECCO2R is not available in the USA. Several trials are currently conducted in Europe.

## Objectives

To assess the use of ECCO2R in France.

## Methods

This retrospective, observational study was performed in French intensive care units (ICUs) from January 2010 to January 2015. A phone interview was conducted with French ICUs affiliated to national societies and public and private hospitals registries. Data recorded were the following: use and indications of ECCO_2_R, type of ECCO_2_R, number of treated patients during the study period, complications associated with the technique, satisfaction rates (in term of efficacy, tolerance and global) based on a scale (0 to 10), and concomitant use of ECMO in the unit.

## Results

222 French ICUs were contacted (52 medical, 20 surgical, 132 polyvalent, 3 cardio-thoracic, 6 paediatric and 2 neurosurgical ICU). Only 3 refused to participate. Thirty-three (15%) ICU had used ECCO_2_R at least once in the past five years, in 292 patients. Most frequent devices used were: iLA^®^ (Novalung) (63%) and Hemolung^®^ (Alung) (36%). The median number per ICU of treated patients was 3[1-7]. The most frequent indication was ultra-protective ventilation for ADRS (54%). Other indications were: failure of NIV during COPD exacerbation (30%), weaning from invasive MV in COPD patients (12%) and miscellaneous (4%). Among ICUs using ECCO_2_R, 22 (67%) reported at least one complication. The most frequent complications were bleeding (45%) and membrane failure (18%). Satisfaction rates were: in term of decarboxylation 7.9 ± 2.4; tolerance 6.9 ± 2.6; overall satisfaction 6.8 ± 2.2. Twenty-one (63%) of the 33 ICUs using ECCO_2_R, also used ECMO. The main reasons for not using ECCO_2_R were the lack of trained staff, unavailability of the device and the lack of scientific evidence (in respectively 56.5%, 38% and 19%).

## Conclusions

These preliminary results show that ECCO_2_R is not widely used in French ICUs. The lack of strong scientific data on outcome is probably the main reason behind the limited use of ECCO_2_R. French studies currently in progress will help define indications of ECCO_2_R and impact on outcome.
